# COGNITIVE STIMULATION THERAPY FOR INDIVIDUALS WITH MEMORY LOSS

**DOI:** 10.1093/geroni/igz038.663

**Published:** 2019-11-08

**Authors:** Max Zubatsky

**Affiliations:** Saint Louis University, Saint Louis, United States

## Abstract

Cognitive Stimulation Therapy (CST) is a non-pharmacological approach for individuals with mild to moderate dementia. It is one of the age friendly community activities of the GWEP to engage older adults with memory challenges. We explored the impact of caregiver-assisted CST (CA-CST) on participants’ overall memory, mood, and retention in the groups. We also investigated the impact of this CST format on the overall well-being of dementia caregivers. Four CST groups (N=28) entered the CA-CST groups meeting once per week for 14 weeks in a university setting. 61 % of participants who completed the group (n=17) showed a .5 point improvement on the Saint Louis University Mental Status Exam (SLUMS), a 2-point decrease on the Cornell Scale for Depression in Dementia (p<.01), and 1.5 point increase in overall caregiver well-being. Continued psychosocial interventions for dementia are needed not just in university settings, but healthcare organizations and other age-friendly settings.

